# Supplementary material for: Homo naledi, a new species of the genus Homo from the Dinaledi Chamber, South Africa
Source: eLife. 2015 Sep 10;4:e09560. doi: 10.7554/eLife.09560 (PMC4559886; doi:10.7554/eLife.09560)
Supplement: Supplementary file 1. — Holotype and paratype specimens and referred materials. DOI: http://dx.doi.org/10.7554/eLife.09560.028 [file elife09560s001.docx]

**Supplementary file 1.** Holotype and paratype specimens and referred materials.

**Holotype specimen**

**DH1**

U.W. 101-1248 Mandibular ramus fragment

U.W. 101-1261 Mandibular corpus with left and right I_1_-M_3_

U.W. 101-1269 M^3^

U.W. 101-1275 Frontal fragment

U.W. 101-1277 Maxilla fragment with left I^1^-M^2^

U.W. 101-1281 Mandibular condyle

U.W. 101-1289 Mandibular ramus fragment

U.W. 101-1290 Parietal fragment

U.W. 101-1303 Fronto-parietal fragment

U.W. 101-1330 Parietal fragment

U.W. 101-1348 Occipital fragment

U.W. 101-1349 Parietal fragment

U.W. 101-1350 Parietal fragment

U.W. 101-1387 Temporal fragment

U.W. 101-1398 Frontal fragment

U.W. 101-1421 Occipital fragment

U.W. 101-1422 Occipital and parietal fragments

U.W. 101-1463 M^3^

U.W. 101-1468 Temporal fragment

U.W. 101-1470 Parietal fragment

U.W. 101-1473 Occipital fragment

**Paratype specimens**

**DH2**

U.W. 101-221 Temporo-parieto-occipital fragment

U.W. 101-906 Frontal fragment

U.W. 101-1111 Frontal fragment

**DH3**

U.W. 101-196 Mandibular condyle

U.W. 101-200 Occipital fragment

U.W. 101-361 Mandibular corpus with left M_2_-M_3_

U.W. 101-396 Parieto-temporal fragment

U.W. 101-419 Frontal fragment

U.W. 101-874 Frontal fragment

**DH4**

U.W. 101-1236 Parieto-occipital fragment

U.W. 101-1296 Temporal fragment

U.W. 101-1297 Occipital fragment

U.W. 101-1299 Parietal fragment

U.W. 101-1372 Temporal fragment

U.W. 101-1582 Temporal (tympanic) fragment

**U.W. 101-377** Mandibular corpus with unworn dentition R_C_-RM_2_

**Referred Material**

**Cranial remains**

U. W. 101-008 zygomatic

U. W. 101-009 frontal fragment

U. W. 101-265 zygomatic

U. W. 101-346 temporal fragment

U. W. 101-409 zygomatic fragment

U. W. 101-521 cranial fragment with temporal and occipital

U. W. 101-546 temporal fragment

U. W. 101-747 temporal fragment

U. W. 101-770 occipital fragment

U. W. 101-1234 occipital fragment

U. W. 101-1252 frontal fragment

U. W. 101-1278 zygomatic

U. W. 101-1354 zygomatic

**Mandibular remains**

U. W. 101-001 mandible with RP_4_-RM_3_

U. W. 101-010 mandible with RC_1_-RP_3_

U. W. 101-325 mandibular condyle

U. W. 101-795 coronoid process

U. W. 101-1001 mandibular ramus fragment

U. W. 101-1142 mandible with RM_2_-RM_3_

**Maxillary Incisors**

U. W. 101-038 LI^1^

U. W. 101-073 RI^2^

U. W. 101-417 LI^2^

U. W. 101-591 LI^2^

U. W. 101-709 LI^2^

U. W. 101-931 RI^1^

U. W. 101-932 RI^2^

U. W. 101-1012 RI^1^

U. W. 101-1558 RI^1^

U. W. 101-1588 LI^2^

U. W. 101-1684 LI^2^

**Maxillary Canines**

U. W. 101-293 C^1?^

U. W. 101-347 LC^1^

U. W. 101-501 LC^1^

U. W. 101-706 LC^1^

U. W. 101-816 RC^1^

U. W. 101-908 RC^1^

U. W. 101-1548 RC^1^

U. W. 101-1556 LC^1^

**Maxillary** **Premolars**

U. W. 101-037 RP^3^

U. W. 101-182 RP^4^

U. W. 101-277 LP^4^

U. W. 101-333 LP^3^

U. W. 101-334 RP^3^

U. W. 101-455 LP^4^

U. W. 101-786 RP^3^

U. W. 101-808 LP^3^

U. W. 101-1004 RP^3^

U. W. 101-1107 LP^4^

U. W. 101-1362 LP^4^ or P^3^

U. W. 101-1401 RP^4^

U. W. 101-1402 RP^3^

U. W. 101-1560 LP^3^

U. W. 101-1561 LP^4^

**Maxillary Molars**

U. W. 101-005 RM^2^

U. W. 101-020 LM^1^

U. W. 101-418 LM^3^

U. W. 101-445 LM^1^

U. W. 101-505 LM^2^

U. W. 101-525 RM^1^

U. W. 101-527 LM^3^

U. W. 101-528 LM^2^

U. W. 101-583 RM^1^

U. W. 101-593 RM^2^

U. W. 101-594 RM^3^

U. W. 101-708 LM^1^

U. W. 101-796 LM^1^

U. W. 101-867 RM^2^

U. W. 101-999 RM^1^

U. W. 101-1002 RM^1^

U. W. 101-1006 RM^2^

U. W. 101-1015 LM^2^

U. W. 101-1063 LM^3?^

U. W. 101-1135 RM^2^

U. W. 101-1305 LM^1^

U. W. 101-1396 RM^1^

U. W. 101-1398 RM^2^ or M^3^

U. W. 101-1463 RM^1^

U. W. 101-1471 LM^2^

U. W. 101-1522 LM^2^

U. W. 101-1676 LM^1^

U. W. 101-1688 RM^1^

**Mandibular Incisors**

U. W. 101-039 RI_1_

U. W. 101-335 RI_2_

U. W. 101-601 LI_1_

U. W. 101-998 LI_2_

U. W. 101-1075 LI_2_

U. W. 101-1131 RI_1_

U. W. 101-1132 RI_2_

U. W. 101-1133 LI_1_

**Mandibular Canines**

U. W. 101-245 RC_1_

U. W. 101-337 RC_1_

U. W. 101-339 RC_1_

U. W. 101-359 LC_1_

U. W. 101-412 LC_1_

U. W. 101-886 RC_1_

U. W. 101-985 LC_1_

U. W. 101-1014 RC_1_

U. W. 101-1076 LC_1_

U. W. 101-1126 LC_1_

U. W. 101-1610 RC_1_

**Mandibular Premolars**

U. W. 101-144 LP_3_

U. W. 101-184 LP_4_

U. W. 101-298 LP_3_

U. W. 101-358 LP_3?_

U. W. 101-383 RP_4_

U. W. 101-506 RP_3_

U. W. 101-729 RP_3_

U. W. 101-800 RP_3_

U. W. 101-850 RP_3_

U. W. 101-887 LP_4_

U. W. 101-889 LP_3_

U. W. 101-1565 LP_3_

**Mandibular Molars**

U. W. 101-006 RM_3_

U. W. 101-145 LM_2_

U. W. 101-284 LM_2_

U. W. 101-285 LM_1_

U. W. 101-297 RM_1_

U. W. 101-344 RM_2_

U. W. 101-507 RM_2_

U. W. 101-516 LM_3_

U. W. 101-582 LM_1_

U. W. 101-589 RM_2?_

U. W. 101-602 RM_1?_

U. W. 101-789 LM_2_

U. W. 101-809 LM_1_

U. W. 101-814 LM_1_

U. W. 101-905 LM_1_

U. W. 101-1304 RM_1_

U. W. 101-1605 LM_2?_

U. W. 101-1689 RM_1_

**Deciduous dentition**

U. W. 101-384 Rdm^2^

U. W. 101-595 Ldc^1^

U. W. 101-655 Rdm_2?_

U. W. 101-728 Rdc^1^

U. W. 101-823 Rdm^1^

U. W. 101-824 Ldc_1_

U. W. 101-1331 Ldi^1^

U. W. 101-1376 Ldm^2^

U. W. 101-1377 Ldm^1^

U. W. 101-1571 Ldc_1_

U. W. 101-1611 Rdc_1_

U. W. 101-1612 Rdi^2^

U. W. 101-1685 Ldm_1_

U. W. 101-1686 Ldm_2_

U. W. 101-1687 Rdm^2^

**Indeterminate dentition**

U. W. 101-388 Root

U. W. 101-680 Root

U. W. 101-652 Germ

U. W. 101-654 Root

U. W. 101-686 Anterior tooth

U. W. 101-864 fragment

U. W. 101-952 incisor

U. W. 101-1086 fragments

U. W. 101-1403 root

U. W. 101-1574 root

U. W. 101-1662 anterior tooth

| **POSTCRANIAL** |  |
| --- | --- |
| **Axial** |  |
| U.W. 101-651 | first cervical vertebra fragment |
| U.W. 101-146 | first cervical vertebra fragment |
| U.W. 101-331 | first cervical vertebra fragment |
| U.W. 101-489/1279 | partial second cervical vertebra |
| U.W. 101-1692 | second cervical vertebra fragment |
| U.W. 101-732 | second cervical vertebra fragment |
| U.W. 101-174 | cervical vertebra fragment |
| U.W. 101-081 | fragment of lower cervical or upper thoracic vertebra |
| U.W. 101-1673 | cervical vertebra fragment (immature) |
| U.W. 101-855 | nearly complete tenth thoracic vertebra |
| U.W. 101-1733 | nearly complete eleventh thoracic vertebra |
| U.W. 101-581 | thoracic vertebra fragment |
| U.W. 101-1614 | thoracic vertebra fragment |
| U.W. 101-663 | thoracic vertebra fragment |
| U.W. 101-015 | thoracic vertebra fragment |
| U.W. 101-575A | thoracic vertebra fragment |
| U.W. 101-515 | centrum of thoracic vertebra |
| U.W. 101-465 | centrum of thoracic vertebra |
| U.W. 101-1302 | thoracic vertebra fragment |
| U.W. 101-439 | thoracic vertebra fragment |
| U.W. 101-1493 | thoracic vertebra fragment (possibly immature) |
| U.W. 101-1054 | thoracic or lumbar vertebra fragment |
| U.W. 101-581 | thoracic vertebra fragment |
| U.W. 101-1669 | thoracic vertebra fragment |
| U.W. 101-1124 | thoracic vertebra fragment |
| U.W. 101-1599 | fragment of lower cervical or upper thoracic vertebra |
| U.W. 101-656 | thoracic vertebra fragment |
| U.W. 101-1693 | thoracic vertebra fragment |
| U.W. 101-478 | partial lumbar vertebra |
| U.W. 101-984 | centrum of lumbar vertebra |
| U.W. 101-1338 | vertebra fragment (possibly lumbar) |
| U.W. 101-1337 | lumbar vertebra fragment |
| U.W. 101-765 | lumbar vertebra fragment |
| U.W. 101-538 | lumbar vertebra fragment |
| U.W. 101-872 | vertebra fragment |
| U.W. 101-1601 | vertebra fragment |
| U.W. 101-1711 | vertebra fragment |
| U.W. 101-362 | vertebra fragment |
| U.W. 101-615 | vertebra fragment |
| U.W. 101-619 | vertebra fragment |
| U.W. 101-673 | vertebra fragment |
| U.W. 101-685 | vertebra fragment |
| U.W. 101-1307 | vertebra fragment |
| U.W. 101-1382 | vertebra fragment |
| U.W. 101-1383 | vertebra fragment |
| U.W. 101-994 | vertebra fragment |
| U.W. 101-847 | vertebra fragment |
| U.W. 101-407 | vertebra fragment |
| U.W. 101-681 | vertebra fragment |
| U.W. 101-1144 | vertebra fragment |
| U.W. 101-1604 | vertebra fragment |
| U.W. 101-323 | vertebra fragment |
| U.W. 101-1615 | vertebra fragment |
| U.W. 101-025 | vertebra fragment |
| U.W. 101-592 | vertebra fragment |
| U.W. 101-620 | vertebra fragment |
| U.W. 101-1621 | vertebra fragment |
| U.W. 101-1699 | vertebra fragment |
| U.W. 101-1003 | vertebra fragment |
| U.W. 101-1079 | vertebra fragment |
| U.W. 101-1397 | vertebra fragment |
| U.W. 101-083 | partial left first rib |
| U.W. 101-621 | left first rib fragment |
| U.W. 101-385 | right second rib fragment |
| U.W. 101-524 | left eleventh rib fragment |
| U.W. 101-1119 | left twelfth rib fragment |
| U.W. 101-1291 | rib fragment |
| U.W. 101-1280 | rib fragment |
| U.W. 101-1090 | rib fragment |
| U.W. 101-979/1009 | rib fragment |
| U.W. 101-1596 | rib fragment |
| U.W. 101-1125 | rib fragment |
| U.W. 101-840 | rib fragment |
| U.W. 101-748 | rib fragment |
| U.W. 101-839 | rib fragment |
| U.W. 101-1670 | rib fragment |
| U.W. 101-657 | rib fragment |
| U.W. 101-079 | rib fragment |
| U.W. 101-479 | rib fragment |
| U.W. 101-456 | rib fragment |
| U.W. 101-951 | rib fragment |
| U.W. 101-351/464/953 | rib fragment |
| U.W. 101-514 | rib fragment |
| U.W. 101-797 | rib fragment |
| U.W. 101-1360 | rib fragment |
| U.W. 101-745 | rib fragment |
| U.W. 101-995 | rib fragment |
| U.W. 101-1569 | rib fragment |
| U.W. 101-1344 | rib fragment |
| U.W. 101-927 | rib fragment |
| U.W. 101-1080 | rib fragment |
| U.W. 101-1109 | rib fragment |
| U.W. 101-509 | rib fragment |
| U.W. 101-978 | rib fragment |
| U.W. 101-669 | rib fragment |
| U.W. 101-024 | rib fragment |
| U.W. 101-764 | rib fragment |
| U.W. 101-1613 | rib fragment |
| U.W. 101-629 | rib fragment |
| U.W. 101-1394 | rib fragment |
| U.W. 101-578 | rib fragment |
| U.W. 101-431 | rib fragment |
| U.W. 101-1059 | rib fragment |
| U.W. 101-392 | rib fragment |
| U.W. 101-1668 | rib fragment |
| U.W. 101-919 | rib fragment |
| U.W. 101-034 | rib fragment |
| U.W. 101-158 | rib fragment |
| U.W. 101-1411 | rib fragment |
| U.W. 101-535 | rib fragment |
| U.W. 101-1472 | rib fragment |
| U.W. 101-437 | rib fragment |
| U.W. 101-273 | rib fragment |
| U.W. 101-1586 | rib fragment |
| U.W. 101-1502 | rib fragment |
| U.W. 101-870 | partial third sternebra |
| U.W. 101-056 | left ilium fragment |
| U.W. 101-450 | left ilium fragment |
| U.W. 101-477 | right ilium fragment |
| U.W. 101-961 | right ilium fragment |
| U.W. 101-986 | right ilium fragment |
| U.W. 101-1100 | right ilium fragment |
| U.W. 101-1249 | left ilium fragment |
| U.W. 101-1370 | left ilium fragment |
| U.W. 101-057 | ilium fragment |
| U.W. 101-058 | ilium fragment |
| U.W. 101-093 | ilium fragment |
| U.W. 101-446 | ilium fragment |
| U.W. 101-476 | ilium fragment |
| U.W. 101-547 | ilium fragment |
| U.W. 101-938 | ilium fragment |
| U.W. 101-1011 | ilium fragment |
| U.W. 101-486 | right ilium fragment (immature) |
| U.W. 101-707 | left ischium fragment |
| U.W. 101-746 | left ischium fragment |
| U.W. 101-791 | right ischium fragment |
| U.W. 101-896 | right ischium fragment |
| U.W. 101-1112 | right ischium fragment |
| U.W. 101-1116 | left ischium fragment |
| U.W. 101-1501 | right ischium fragment |
| U.W. 101-1228 | ischium fragment |
| U.W. 101-1603 | ischium fragment |
| U.W. 101-1088 | right ischium fragment (immature) |
| U.W. 101-1414 | left ischium fragment (immature) |
| U.W. 101-1245 | left pubis fragment |
| U.W. 101-1250 | right pubis fragment |
| U.W. 101-842 | left pubis fragment |
| U.W. 101-1616 | right pubis fragment |
| U.W. 101-1700 | right pubis fragment |
| U.W. 101-256 | pubis fragment |
| U.W. 101-1661 | pubis fragment |
| U.W. 101-S1428 | left pubis fragment (immature) |
| U.W. 101-723 | sacrum fragment (immature) |
| U.W. 101-888 | coccyx fragment (immature) |
|  |  |
| **Pectoral girdle and upper limb** | |
| U.W. 101-036 | right clavicular shaft (possibly immature) |
| U.W. 101-258 | right clavicular shaft |
| U.W. 101-895 | clavicle fragment |
| U.W. 101-1083 | left clavicle fragment |
| U.W. 101-1229 | right clavicle fragment (possibly immature) |
| U.W. 101-1256 | clavicle fragment |
| U.W. 101-1347 | left clavicle fragment |
| U.W. 101-1503 | left clavicle fragment |
| U.W. 101-1521 | right clavicle fragment |
| U.W. 101-032 | scapular spine fragment |
| U.W. 101-243 | partial left scapula |
| U.W. 101-462 | scapular spine fragment |
| U.W. 101-666 | left scapula fragment |
| U.W. 101-820 | left scapula fragment |
| U.W. 101-834 | scapula fragment |
| U.W. 101-1270 | scapula fragment |
| U.W. 101-1276 | scapula fragment |
| U.W. 101-1301 | partial right scapula |
| U.W. 101-1353 | scapular spine fragment (possibly immature) |
| U.W. 101-1540 | right scapula fragment |
| U.W. 101-031 | right distal humerus (immature) |
| U.W. 101-276 | left distal humerus |
| U.W. 101-283 | right humerus |
| U.W. 101-411 | humeral shaft fragment (possibly immature) |
| U.W. 101-458 | right humeral shaft fragment |
| U.W. 101-466 | right distal humerus |
| U.W. 101-650 | proximal humerus fragment |
| U.W. 101-739 | right humeral shaft fragment |
| U.W. 101-744 | left distal humerus |
| U.W. 101-749 | right proximal humerus fragment |
| U.W. 101-948 | right humerus (immature) |
| U.W. 101-1208 | humerus fragment |
| U.W. 101-1216 | humerus fragment |
| U.W. 101-1219 | humerus fragment |
| U.W. 101-1240 | right distal humerus |
| U.W. 101-1340 | humeral shaft fragment (possibly immature) |
| U.W. 101-1490 | proximal humerus fragment |
| U.W. 101-1542 | distal humerus fragment |
| U.W. 101-1710 | proximal humerus fragment |
| U.W. 101-016 | ulnar shaft fragment |
| U.W. 101-022 | ulnar shaft fragment |
| U.W. 101-061 | left distal ulna fragment |
| U.W. 101-075 | proximal ulna fragment |
| U.W. 101-142 | left distal ulna fragment |
| U.W. 101-320 | right proximal ulnar shaft fragment |
| U.W. 101-348 | left proximal ulnar shaft fragment |
| U.W. 101-430 | ulnar shaft fragment |
| U.W. 101-499 | right ulnar shaft fragment |
| U.W. 101-560 | right proximal ulnar fragment |
| U.W. 101-600 | left proximal ulnar fragment (possibly immature) |
| U.W. 101-714 | distal ulna fragment |
| U.W. 101-830 | ulnar shaft fragment |
| U.W. 101-831 | right distal ulna fragment |
| U.W. 101-964 | right proximal ulna fragment |
| U.W. 101-967 | ulna fragment |
| U.W. 101-1043 | right proximal ulna fragment |
| U.W. 101-1239 | ulnar shaft fragment |
| U.W. 101-1293 | ulnar shaft fragment (possibly immature) |
| U.W. 101-1378 | right distal ulna fragment |
| U.W. 101-1480 | right ulnar shaft fragment |
| U.W. 101-1517 | right proximal ulna fragment |
| U.W. 101-1566 | ulnar shaft fragment |
| U.W. 101-1609 | ulnar shaft fragment |
|  |  |
| **Hand** |  |
| *left-side antimeres of elements in Hand 1 (paratype)* | |
| U.W. 101-033 | left proximal Mc3 (antimere to U.W. 101-1319 of hand 1) |
| U.W. 101-418B | left lunate (antimere to U.W. 101-1732 of hand 1) |
| U.W. 101-1464 | left PP3 (antimere to U.W. 101-1327 hand 1) |
| *Hand 2, adult (comprised of both right- and left-side elements, associations tentative)* | |
| U.W. 101-713 | partial left hamate (antimere to U.W. 101-1640) |
| U.W. 101-807 | left scaphoid (antimere to U.W. 101-1639) |
| U.W. 101-1282 | left Mc1 (antimere to U.W. 101-1641) |
| U.W. 101-1454 | left PP5 (antimere to U.W. 101-1645) |
| U.W. 101-1466 | left proximal Mc5 |
| U.W. 101-1639 | right scaphoid (antimere to U.W. 101-807) |
| U.W. 101-1640 | right hamate (antimere to U.W. 101-713) |
| U.W. 101-1641 | right Mc1 (antimere to U.W. 101-1282) |
| U.W. 101-1642 | right PP1 (pathological) |
| U.W. 101-1643 | PP3, possibly left |
| U.W. 101-1644 | PP4, possibly right |
| U.W. 101-1645 | right PP5 (antimere to U.W. 101-1454) |
| U.W. 101-1646 | IP3 |
| U.W. 101-1647 | IP4 |
| U.W. 101-1648 | IP5 |
| U.W. 101-1649 | non-pollical DP |
| U.W. 101-1650 | right Mc2 shaft |
| U.W. 101-1651/1628 | right Mc3 |
| U.W. 101-1653 | Mc shaft fragment |
| U.W. 101-1655 | phalanx fragment |
| U.W. 101-1656 | scaphoid fragment |
| *Hand 3, adult (comprised of both right- and left-side elements, associations tentative)* | |
| U.W. 101-270 | right Mc1 |
| U.W. 101-916 | left trapezium |
| U.W. 101-930 | left capitate (antimere to U.W. 101-1385) |
| U.W. 101-1385 | right capitate (antimere to U.W. 101-930) |
| *Hand 4, immature (comprised of both right- and left-side elements)* | |
| U.W. 101-559 | left Mc3 |
| U.W. 101-517/721 | right Mc3 |
| *Hand 5, immature* | |
| U.W. 101-1029 | Mc shaft fragment |
| U.W. 101-1271 | Mc shaft fragment |
| U.W. 101-1536 | Mc shaft fragment |
| U.W. 101-1633 | Mc shaft fragment |
| U.W. 101-1635 | partial PP |
| U.W. 101-1636 | Mc shaft fragment |
| U.W. 101-1654 | Mc shaft fragment |
| U.W. 101-1664 | distal IP |
| *Isolated hand bones* | |
| U.W. 101-007 | left Mc1 |
| U.W. 101-036 | Mc shaft fragment |
| U.W. 101-120 | left PP1 fragment |
| U.W. 101-168 | PP shaft fragment |
| U.W. 101-175 | partial PP |
| U.W. 101-178 | IP (IP5?) |
| U.W. 101-381 | IP |
| U.W. 101-400 | partial left Mc2 |
| U.W. 101-401 | left Mc1 shaft |
| U.W. 101-418A | Mc shaft fragment |
| U.W. 101-428 | left PP1 |
| U.W. 101-512 | right proximal Mc2 |
| U.W. 101-537 | Mc fragment |
| U.W. 101-554 | partial PP |
| U.W. 101-558 | PP (PP3 or PP4?) |
| U.W. 101-603 | partial IP |
| U.W. 101-604 | non-pollical DP |
| U.W. 101-665 | IP |
| U.W. 101-678 | phalangeal shaft fragment |
| U.W. 101-720 | proximal PP1 |
| U.W. 101-754 | PP (PP2 or PP4?) |
| U.W. 101-757 | left distal Mc2 |
| U.W. 101-777 | IP (IP5?) |
| U.W. 101-868 | metapodial shaft fragment |
| U.W. 101-913 | partial PP (PP3 or PP4?) |
| U.W. 101-917 | left Mc1 |
| U.W. 101-923 | PP |
| U.W. 101-924 | IP (IP3 or IP4?) |
| U.W. 101-982 | IP shaft fragment |
| U.W. 101-1025 | PP |
| U.W. 101-1027 | IP (IP3 or IP4?) |
| U.W. 101-1055 | left PP1 |
| U.W. 101-1225 | Mc shaft fragment |
| U.W. 101-1232 | Mc shaft fragment (Mc2L?) |
| U.W. 101-1237 | immature PP |
| U.W. 101-1247/1630 | PP (PP5?) |
| U.W. 101-1264 | Mc shaft fragment (Mc3?) |
| U.W. 101-1265 | Mc shaft fragment |
| U.W. 101-1298 | left proximal Mc2 |
| U.W. 101-1348 | PP |
| U.W. 101-1365 | Mc shaft fragment |
| U.W. 101-1379 | IP |
| U.W. 101-1380 | PP shaft |
| U.W. 101-1424 | PP (PP3?) |
| U.W. 101-1440 | IP (IP5?) |
| U.W. 101-1453 | right DP1 |
| U.W. 101-1455 | IP (IP3 or IP4?) |
| U.W. 101-1460 | PP (PP2 or PP4?) |
| U.W. 101-1462 | left distal Mc2 fragment |
| U.W. 101-1474 | right proximal Mc2 |
| U.W. 101-1478 | PP (PP5?) |
| U.W. 101-1479 | IP |
| U.W. 101-1516 | PP shaft fragment, possibly immature |
| U.W. 101-1527 | non-pollical DP |
| U.W. 101-1539 | partial PP |
| U.W. 101-1541 | Mc shaft fragment |
| U.W. 101-1545 | partial right trapezoid |
| U.W. 101-1546 | right lunate |
| U.W. 101-1552 | PP epiphysis |
| U.W. 101-1580 | partial right trapezium |
| U.W. 101-1581 | right trapezoid |
| U.W. 101-1582 | non-pollical DP |
| U.W. 101-1583 | Mc head |
| U.W. 101-1590 | non-pollical DP |
| U.W. 101-1607 | non-pollical DP |
| U.W. 101-1619 | IP |
| U.W. 101-1620 | PP shaft fragment |
| U.W. 101-1624 | right scaphoid fragment |
| U.W. 101-1631 | Mc shaft fragment |
| U.W. 101-1632 | Mc shaft fragment |
| U.W. 101-1634 | Mc shaft fragment |
| U.W. 101-1637 | IP shaft fragment |
| U.W. 101-1652 | Mc shaft fragment |
| U.W. 101-1658 | Mc head |
| U.W. 101-1660 | IP (IP2 or IP4?) |
| U.W. 101-1702 | triquetrum |
| U.W. 101-1703 | non-pollical DP |
| U.W. 101-1704 | IP |
| U.W. 101-1705 | IP |
| U.W. 101-1706 | IP |
| U.W. 101-1708 | PP fragment |
| U.W. 101-1709 | PP fragment |
| U.W. 101-1715 | Mc head (immature) |
| U.W. 101-1718 | non-pollical DP |
| U.W. 101-1748 | Mc shaft fragment |
| U.W. 101-1749 | proximal right Mc3 shaft fragment |
| U.W. 101-1750 | metapoidal head fragment |
|  | |
| **Lower limb** | |
| U.W. 101-002 | right proximal femur |
| U.W. 101-003 | right femoral shaft |
| U.W. 101-012 | right femoral shaft |
| U.W. 101-014 | partial femoral shaft (possibly immature) |
| U.W. 101-018 | right proximal femur |
| U.W. 101-143 | right proximal femoral shaft |
| U.W. 101-215 | left distal femur |
| U.W. 101-268 | left femoral shaft |
| U.W. 101-271 | femoral head |
| U.W. 101-341 | femoral shaft fragment |
| U.W. 101-398/226 | left partial proximal femur |
| U.W. 101-421 | left partial proximal femur |
| U.W. 101-545 | right femoral shaft |
| U.W. 101-857 | left distal femoral shaft fragment |
| U.W. 101-863 | left proximal femur (immature) |
| U.W. 101-898 | femoral condylar fragment |
| U.W. 101-938 | right partial proximal femur (immature) |
| U.W. 101-1000 | right partial proximal femur (immature) |
| U.W. 101-1120 | partial distal femur (immature) |
| U.W. 101-1136 | right partial proximal femur |
| U.W. 101-1284 | right distal femoral shaft fragment |
| U.W. 101-1300 | femoral head |
| U.W. 101-1434 | left proximal femoral shaft fragment |
| U.W. 101-1475 | left partial proximal femur |
| U.W. 101-1482 | left femoral shaft fragment |
| U.W. 101-1555 | right femoral neck fragment (immature) |
| U.W. 101-852 | right patella |
| U.W. 101-1404 | right patella |
| U.W. 101-1512 | partial patella |
| U.W. 101-1639 | right patella |
| U.W. 101-013 | distal tibial shaft fragment |
| U.W. 101-017 | right proximal tibial shaft fragment |
| U.W. 101-042 | tibial shaft fragment |
| U.W. 101-072 | left tibial shaft fragment |
| U.W. 101-085 | tibial shaft fragment |
| U.W. 101-136 | right proximal tibial shaft fragment |
| U.W. 101-213 | right proximal tibial shaft fragment |
| U.W. 101-237 | left proximal tibial shaft fragment |
| U.W. 101-239 | proximal tibial shaft fragment |
| U.W. 101-313 | left proximal tibial shaft fragment |
| U.W. 101-402 | right distal tibial shaft fragment |
| U.W. 101-420 | left distal tibia |
| U.W. 101-498 | left tibial shaft fragment |
| U.W. 101-500 | left tibial shaft fragment |
| U.W. 101-567 | tibial shaft fragment |
| U.W. 101-571 | right proximal tibial shaft |
| U.W. 101-586 | tibial shaft fragment |
| U.W. 101-711 | left distal tibia |
| U.W. 101-848 | right tibial shaft fragment |
| U.W. 101-973 | left tibial shaft fragment |
| U.W. 101-996/1074/1077 | right tibia (immature) |
| U.W. 101-1070 | left tibia (immature) |
| U.W. 101-1210 | right distal tibial shaft fragment |
| U.W. 101-1214 | right midshaft tibial shaft fragment |
| U.W. 101-1220 | tibial shaft fragments |
| U.W. 101-1241 | left distal tibial shaft fragment |
| U.W. 101-1262 | right distal tibia |
| U.W. 101-1288 | right distal tibial shaft fragment |
| U.W. 101-1295 | tibial shaft fragment |
| U.W. 101-1481 | left distal tibial shaft fragment |
| U.W. 101-1518 | left distal tibia fragment |
| U.W. 101-181 | distal fibular shaft fragment |
| U.W. 101-416 | left proximal fibular shaft fragment |
| U.W. 101-449 | proximal fibular shaft fragment |
| U.W. 101-508 | right midshaft fibular shaft fragment |
| U.W. 101-580 | left distal fibular shaft fragment |
| U.W. 101-675 | proximal fibula fragment |
| U.W. 101-702 | left proximal fibular shaft fragment |
| U.W. 101-719 | left proximal fibular shaft fragment |
| U.W. 101-722 | proximal fibular fragment |
| U.W. 101-737/774 | right distal fibular shaft fragment |
| U.W. 101-778 | distal fibular shaft fragment |
| U.W. 101-782 | distal fibular shaft fragment |
| U.W. 101-806 | fibular shaft fragment |
| U.W. 101-813 | fibular shaft fragment |
| U.W. 101-817 | partial proximal fibula (immature) |
| U.W. 101-876 | proximal fibular shaft fragment |
| U.W. 101-892 | distal fibular shaft fragment |
| U.W. 101-902 | left proximal fibular shaft fragment |
| U.W. 101-925 | right proximal fibular shaft fragment |
| U.W. 101-968 | right distal fibular shaft fragment |
| U.W. 101-987 | proximal fibular shaft fragment |
| U.W. 101-1037/1498 | left fibular shaft |
| U.W. 101-1045 | left partial fibula (immature) |
| U.W. 101-1046 | left distal fibular shaft fragment |
| U.W. 101-1066 | midshaft fibular shaft fragment |
| U.W. 101-1071 | distal fibular shaft fragment |
| U.W. 101-1094 | fibular shaft fragment |
| U.W. 101-1113 | right proximal fibula |
| U.W. 101-1114 | distal fibular shaft fragment |
| U.W. 101-1115 | midshaft fibular shaft fragment |
| U.W. 101-1122 | fibular shaft fragment |
| U.W. 101-1138 | proximal fibular shaft fragment |
| U.W. 101-1143 | right fibular shaft fragment |
| U.W. 101-1231 | distal fibula fragment |
| U.W. 101-1254 | right proximal fibular shaft fragment |
| U.W. 101-1259 | fibular shaft fragment |
| U.W. 101-1260 | left proximal fibular shaft fragment |
| U.W. 101-1313 | proximal fibular shaft fragment |
| U.W. 101-1436 | midshaft fibular shaft fragment |
| U.W. 101-1437 | right distal fibular epiphysis |
| U.W. 101-1451 | left proximal fibular shaft fragment |
| U.W. 101-1520 | fibular shaft fragment (possibly immature) |
| U.W. 101-1679 | right proximal fibular shaft fragment |
| U.W. 101-1701 | left distal fibula |
|  |  |
| **Foot** |  |
| U.W. 101-080 | left talus (immature) |
| U.W. 101-148/149 | left talus |
| U.W. 101-520 | left talus |
| U.W. 101-910 | left talus (immature) |
| U.W. 101-1031 | left talus |
| U.W. 101-1215 | left talus fragment |
| U.W. 101-1623 | right talus (immature) |
| U.W. 101-724 | left calcaneus |
| U.W. 101-907 | left calcaneus (immature) |
| U.W. 101-1662 | right calcaneus fragment (immature) |
| U.W. 101-623 | right navicular fragment |
| U.W. 101-811 | left navicular |
| U.W. 101-910 | left navicular (immature) |
| U.W. 101-997 | right navicular (immature?) |
| U.W. 101-1030 | left navicular |
| U.W. 101-1039 | left medial cuneiform |
| U.W. 101-1062 | left medial cuneiform |
| U.W. 101-1535 | left medial cuneiform |
| U.W. 101-1242 | right intermediate cuneiform (immature) |
| U.W. 101-1457 | right intermediate cuneiform |
| U.W. 101-1534 | left intermediate cuneiform |
| U.W. 101-1618 | left intermediate cuneiform |
| U.W. 101-1682 | left intermediate cuneiform (immature?) |
| U.W. 101-1695 | right intermediate cuneiform |
| U.W. 101-683 | left lateral cuneiform |
| U.W. 101-1734 | left lateral cuneiform |
| U.W. 101-487 | right cuboid (immature) |
| U.W. 101-1023 | left cuboid |
| U.W. 101-244 | left Mt1 (immature) |
| U.W. 101-496 | left Mt1 |
| U.W. 101-1019 | left Mt1 |
| U.W. 101-1530 | right Mt1 |
| U.W. 101-1499 | right Mt1 epiphysis (immature) |
| U.W. 101-459/461 | right Mt2 |
| U.W. 101-1022 | left Mt2 |
| U.W. 101-1499 | right Mt2 (immature) |
| U.W. 101-552 | left Mt3 |
| U.W. 101-1035 | left Mt3 |
| U.W. 101-1500 | right Mt3 (immature) |
| U.W. 101-248 | left Mt4 (immature) |
| U.W. 101-269 | right Mt4 |
| U.W. 101-1368 | right Mt4 (immature) |
| U.W. 101-518 | right Mt5 |
| U.W. 101-1412 | right Mt5 |
| U.W. 101-497 | Mt shaft fragment |
| U.W. 101-750 | Mt shaft fragment |
| U.W. 101-801 | Mt head fragment |
| U.W. 101-869 | Mt head fragment |
| U.W. 101-1437 | Mt shaft fragments |
| U.W. 101-1444 | Mt shaft |
| U.W. 101-1513 | Mt shaft |
| U.W. 101-1559 | Mt shaft |
| U.W. 101-1585 | Mt shaft |
| U.W. 101-082 | left PP1 |
| U.W. 101-504 | left PP |
| U.W. 101-725 | PP head and shaft |
| U.W. 101-976 | PP |
| U.W. 101-1013 | left PP |
| U.W. 101-1024 | left PP1 |
| U.W. 101-1034 | left PP |
| U.W. 101-1148 | PP |
| U.W. 101-1359 | PP |
| U.W. 101-1441 | PP |
| U.W. 101-1442 | PP1 |
| U.W. 101-1452 | PP1 |
| U.W. 101-1557 | PP |
| U.W. 101-1657 | PP (immature) |
| U.W. 101-550 | IP |
| U.W. 101-661 | IP |
| U.W. 101-665 | IP |
| U.W. 101-988 | IP |
| U.W. 101-1042 | IP |
| U.W. 101-1399 | IP |
| U.W. 101-1438 | IP |
| U.W. 101-1484 | IP |
| U.W. 101-1549 | IP |
| U.W. 101-1575 | IP |
| U.W. 101-1587 | IP |
| U.W. 101-1591 | IP |
| U.W. 101-1594 | IP |
| U.W. 101-1625 | IP |
| U.W. 101-988 | DP |
| U.W. 101-1010 | DP |
| U.W. 101-1526 | DP |
| U.W. 101-1527 | DP |
| U.W. 101-1550 | DP |
| U.W. 101-1576 | DP |
| U.W. 101-884 | phalangeal fragment |
| U.W. 101-1118 | phalangeal fragment |
| U.W. 101-1589 | phalangeal fragment |
| U.W. 101-1592 | phalangeal fragment |
| U.W. 101-1595 | phalangeal fragment |
| U.W. 101-1598 | phalangeal fragment |
